# Cardiac Nonmyocyte Cell Functions and Crosstalks in Response to Cardiotoxic Drugs

**DOI:** 10.1155/2017/1089359

**Published:** 2017-10-22

**Authors:** Jessica Gambardella, Bruno Trimarco, Guido Iaccarino, Daniela Sorriento

**Affiliations:** ^1^Dipartimento di Medicina e Chirurgia, Università degli Studi di Salerno, via Salvatore Allende, 84081 Baronissi, Italy; ^2^Dipartimento di Scienze Biomediche Avanzate, Università Federico II di Napoli, via Pansini 5, 80131 Napoli, Italy

## Abstract

The discovery of the molecular mechanisms involved in the cardiac responses to anticancer drugs represents the current goal of cardio-oncology research. The oxidative stress has a pivotal role in cardiotoxic responses, affecting the function of all types of cardiac cells, and their functional crosstalks. Generally, cardiomyocytes are the main target of research studies on cardiotoxicity, but recently the contribution of the other nonmyocyte cardiac cells is becoming of growing interest. This review deals with the role of oxidative stress, induced by anticancer drugs, in cardiac nonmyocyte cells (fibroblasts, vascular cells, and immune cells). The alterations of functional interplays among these cardiac cells are discussed, as well. These interesting recent findings increase the knowledge about cardiotoxicity and suggest new molecular targets for both diagnosis and therapy.

## 1. Introduction

Cardiotoxicity is a severe adverse reaction of the heart in response to anticancer drugs; this “toxicity that affects the heart,” as defined by the National Cancer Institute, includes both direct effects on cardiac muscle and indirect effects caused by hemodynamic alterations or thrombotic events. Cardiotoxic-derived phenotypes are highly variable in terms of onset and severity of the disease. Indeed, early and late cardiac events can occur after antineoplastic therapy and may range from subclinical dysfunction to irreversible heart failure. Typical cardiac responses to antineoplastic therapy are left ventricular dysfunction, ischemia, or rhythm disturbances [1]. This high variability is not always related to the drug (type, dose, and duration of administration). Indeed, the cardiotoxic response is also associated with other factors, such as genetic predisposition, systemic inflammatory status, cardiovascular risk factors, and preexisting cardiac dysfunction [2]. This complexity makes the clinical management very challenging and negatively affects the patient outcome [3, 4].

The current cardio-oncology research is going after the possibility to avoid or reduce the cardiotoxic effects of antitumoral agents, to enhance the oncologic benefits of the therapy and the identification of early markers of heart failure, and to promptly start an effective therapy. To date, several molecules have been suggested to be useful biomarkers of cardiotoxicity, such as cardiac troponin and natriuretic peptides. Indeed, their serum levels increase in response to anticancer therapy in a dose-dependent manner and correlate with the degree of left ventricular dysfunction [5, 6]. Moreover, other molecular markers of cardiotoxicity have been proposed, such as FABP, GPBB, hs-CRP, IL-6, and MPO, even if further studies are needed to better clarify their role in such phenomenon [7]. Therefore, it is essential to understand the molecular mechanisms that are involved in cardiotoxicity to identify potential biomarkers and therapeutic agents.

The leading mechanistic hypothesis for drug-induced cardiotoxicity is the increase of reactive oxygen species (ROS) within the cardiac myocyte, which are highly toxic and can cause direct damage to proteins, lipids, and DNA. Indeed, antineoplastic agents can directly act as a source of reactive species (anthracycline) or indirectly mediate metabolic dysfunction or reduce the antioxidant capacity of the cell [8]. ROS accumulation affects several cellular functions, such as apoptosis, senescence, proliferation, cellular migration, infiltration, and inflammation, all of which contribute to cardiotoxicity [9, 10]. Moreover, ROS are able to induce latent effects that lead to progressive alterations of DNA, mitochondrial DNA, and cellular membranes [11, 12]. For this reason, ROS have been proposed as “molecular keepers” of metabolic memory that sustain an ongoing damage, creating a state of susceptibility [13]. Adult myocytes are more susceptible to these drugs because myocytes are terminally differentiated and cannot sufficiently replicate in order to replace cells damaged during treatment. However, cellular alterations induced by ROS in response to anticancer drugs can also affect other cellular components of the heart, such as endothelial cells, cardiac fibroblasts, vascular smooth muscle cells, and their functional crosstalk. In this review, we deal with the effects of oxidative stress induced by anticancer drugs in cardiac nonmyocyte cells. This focus is becoming of great relevance since cardiac nonmyocyte cells have a key role in response to pathological stimuli, such as oxidative stress, and the study of their functional interactions may offer the possibility to identify new therapeutic targets for cardiotoxicity.

## 2. Anticancer Drugs Inducing Oxidative Stress

Anticancer drugs that exert cardiotoxic effects are numerous and can be divided into two classes depending on their direct or indirect effect on ROS production (Table 1). The first class of drugs comprises the ones that have a direct effect on the generation of ROS production, including anthracyclines, cyclophosphamide, 5-fluorouracil, and cisplatinum. To date, the anthracycline-containing regimen, including cyclophosphamide, fluorouracil, and/or paclitaxel/docetaxel, represents the most adopted life-saving chemotherapy scheme in the treatment of several cancers, such as breast cancer [14] [15]. Despite the effectiveness as anticancer agents, the use of anthracyclines, such as doxorubicin (DOX), is limited due to their cardiotoxic effects, which can lead to severe heart failure and death. Several studies demonstrate that these toxic effects on the heart are directly associated with their ability to generate ROS and, in particular, to trigger a vicious circle of ROS production [16, 17]. The reaction starts with a reduction of doxorubicin by NADPH-cytP450 reductase that results in semiquinone radical formation; this doxoradical reacts with iron, inducing the formation of the complex anthracycline-iron (Fe^2+^) [18]. This complex is able to react with and reduce oxygen, producing superoxide and regenerating doxorubicin, thus triggering a vicious circle of ROS production. DOX is also able to bind the endothelial nitric oxide synthase, eNOS, leading to an increase of superoxide and a reduction of nitric oxide synthesis, that negatively affect the cardiovascular homeostasis [19]. Moreover, DOX causes further reduction of the antioxidant capacity of the heart, inducing a global high cardiac oxidative stress [20]. Although other classes of anticancer drugs also affect the oxidant balance, ROS production in response to anthracyclines is the most studied and better characterized. Indeed, also cyclophosphamide (CP) is able to generate reactive metabolite but the involved molecular mechanism is not fully delineated. CP is an anticancer drug widely used in the treatment of several types of cancer, such as leukemia, ovarian, and breast cancer, and also in association with anthracyclines [21]. It has been shown that the cardiotoxic effects of CP are due to the accumulation of mitochondrial superoxide radicals that lead to the damage of the inner mitochondrial membrane resulting in the increase of calcium permeability and rapidly impairment of cellular respiration [22].

The antimetabolite 5-fluorouracil (5-FU) is also able to induce alterations of oxidative balance. It is widely used as an adjuvant drug in the treatment of colon, pancreas, and liver cancer [23]. In vitro studies show that the exposition to 5-FU induces a dose- and time-dependent ROS production in both endothelial and cardiac cells [24, 25].

The association between oxidative damage and toxicity was observed also in the case of cytostatic drug administration, such as cisplatinum. A diffuse oxidative damage in several tissues was observed *in vivo* after exposure to this drug [26, 27]. It has been demonstrated that ROS generation occurs in the mitochondria due to the impairment of protein synthesis and that ROS-related cytotoxicity of cisplatinum varies depending on the mitochondrial redox status [28].

A second class of drugs, including taxanes, trastuzumab, and sorafenib, indirectly affects redox balance. Indeed, they are able to enhance the oxidative stress induced by other chemotherapeutics or to affect key pathways involved in cellular responses to oxidative damage. Taxanes, such as paclitaxel, usually used in combination with anthracycline, potentiate the toxic effects of DOX by increasing its plasma levels and enhancing the production of DOX-reactive metabolite in the heart [29].

A target-specific anticancer drug, trastuzumab, induces cardiotoxic effects by affecting survival pathways. Indeed, it inhibits heregulin-HER signaling thus protecting the cells from apoptosis induced by oxidative stress [30, 31]. A similar mechanism is also activated by the antiproliferative and antiangiogenetic drug sorafenib. In fact, its toxicity seems to be related to its inhibitory effect on RAF1. RAF1 is able to block two proapoptotic kinases, ASK1 and MST2, important in ROS-induced damage [32]. Therefore, sorafenib-dependent RAF1 inhibition promotes apoptosis in response to oxidative stress [8].

In summary, ROS-induced cellular injury represents a common off-target effect of many anticancer drugs, with cardiotoxic activity. It is known that ROS have a key role in the development of cardiovascular diseases since they affect several cellular processes, such as cellular migration, proliferation, hypertrophy, angiogenesis, apoptosis, and senescence [33]. The most common drugs used in the treatment of cardiovascular diseases exert a double effect, regulating not only the adrenergic receptor activation but also ROS production. Indeed, *β*-blockers, such as carvedilol and nebivolol, showed significant cardiac protection in patients under anthracycline treatment [34]. Besides their effect on adrenoceptors, carvedilol is able to reduce ROS formation in doxorubicin-treated cardiomyocytes [35], whereas nebivolol prevents the generation of peroxynitrite and nitric oxide synthase uncoupling [36].

## 3. Cellular Targets in the Heart

It has been estimated that the adult murine heart, approximately, consists of 56% of myocytes, 27% of fibroblasts, 7% of endothelial cells, and 10% of vascular smooth muscle cells and immune cells [37]. All these cardiac cells are functionally related and cooperate to guarantee the appropriate cardiac output. Indeed, if cardiomyocytes generate the contractile force, the fibroblasts produce essential components of extracellular matrix ensuring a functional cardiac architecture, and the endothelial cells act as a functional barrier of the vessels, regulating not only the fluxes of nutrients and oxygen but also the flux of xenobiotic from circulation toward the heart [38–40]. Cardiac endothelial cells also release paracrine factors that regulate cardiomyocyte metabolism, survival, and contractile function [41]. Several studies demonstrate that all cell types in the heart are involved in ROS production, in response to anticancer drugs (mainly doxorubicin), contributing to the cardiotoxic phenotype (Table 2). Here, we report an overview of nonmyocyte cardiac cells which are affected by anticancer drug-induced oxidative stress and contribute to the development of cardiotoxicity.

### 3.1. Cardiac Fibroblasts

**Figure 1:**
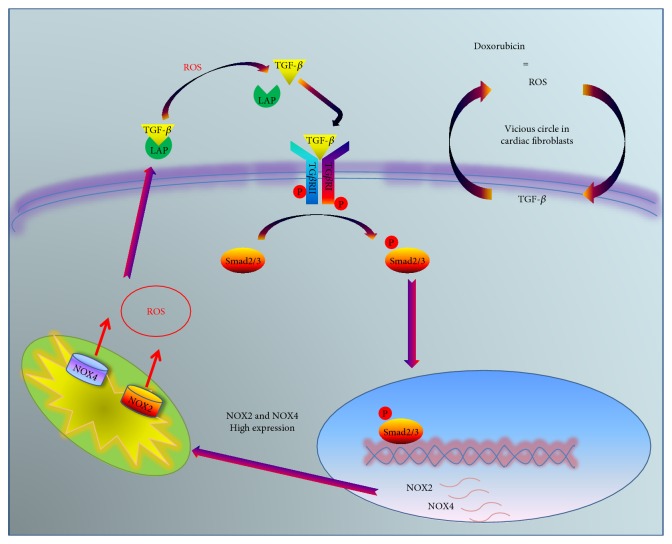
The vicious cycle of ROS production in cardiac fibroblast. DOX induces ROS production that mediates the activation of TGF-*β* through its release from LAP. Through its paracrine action, TGF-*β* induces expression of genes involved in oxidative stress, NOX2, and NOX4, promoting the further increase of ROS.

It has been recently demonstrated that cardiac fibroblasts are involved in the processes of cardiac remodeling after stress [42]. Moreover, a role has been identified for these cells in oxidative stress induced by chemotherapeutics and, in particular, by doxorubicin. DOX induces accumulation of fibrotic tissue as a result of excessive ROS generation; in particular, in cardiac fibroblast, DOX induces ROS-dependent activation of TGF-*β*, resulting in the pathological deposition of collagen [43, 44]. Then, DOX-dependent ROS trigger a vicious circle that deteriorates this condition: ROS activate TGF-*β*, and this latter, in turn, activates further ROS production [45] (Figure 1). It is known that TGF-*β* is produced as an inactive precursor complex including the latency-associated peptide (LAP) [46]. The interaction with LAP can be disrupted not only by the specific proteolytic process but also by chemical and physical conditions, such as heat, acidification, and oxidation of LAP by ROS exposure [47, 48]. Therefore, in cardiac fibroblasts, the high levels of ROS, produced by DOX metabolism, induce LAP dissociation and the release of active TGF-*β*. The active form of TGF-*β* in turn increases NOX4 and NOX2 gene expression resulting in a further increase of ROS generation [49]. Furthermore, TGF-*β* mediates the downregulation of antioxidant enzymes, such as catalase and glutathione peroxidase [50], thus favoring oxidative stress. Therefore, in this context, cardiac fibroblasts act as “amplifiers” of oxidative stress induced by DOX exposure. Accordingly, cardiac fibroblasts isolated from DOX-treated rats are characterized by high levels of TGF-*β* and a profibrotic phenotype [51]. Interestingly, these fibroblasts conserve these features also *in vitro* after several passages, enforcing the putative role of ROS as the “molecular keepers” of metabolic memory, and able to confer a “signature” to the stressed cells that are also transmitted to the progeny.

Apart from TGF-*β*, another protein is regulated in cardiac fibroblasts in response to DOX, the oxidative stress-sensing molecule, ATM protein. ATM is a kinase that is recruited and activated in response to oxidative stress-induced DNA damage [52]. Interestingly, ATM typical response to oxidative stress occurs only in cardiac fibroblasts after DOX exposure and not in cardiomyocytes. Indeed, mice with selective deletion of ATM in fibroblasts were protected from DOX-induced cardiotoxicity, compared with cardiomyocyte-specific knockout mice [53]. These findings are confirmed in an *in vitro* study showing that even if the aconitase activity in basal condition is higher in fibroblasts than in cardiomyocytes, fibroblasts also have the highest aconitase inactivation after DOX, resulting in cell mortality [54].

Cardiac fibroblasts seem to have a role also in cardiotoxic response to radiation, in particular to cobalt. In fact, it is ascertained that cobalt affects the contractile function of the heart [55]. In rats exposed to cobalt, there is a strong accumulation of cobalt that leads to a significant reduction of Mn-SOD activity, suggesting that the isotope is able to reduce the intrinsic ROS scavenger capacity [55]. This effect is due to the effects of cobalt on both cardiac myocytes and fibroblasts. In vivo, this could lead to morphological alterations of the heart due to reduced matrix deposition and the altered interplay between contractile cells and fibroblasts which cause a strong impairment of contractility.

All these findings suggest that, given their ability to amplify the oxidative signal, fibroblasts could be the first cellular target of oxidative stress induced by anticancer drugs, which could be then able to trigger cardiomyocyte dysfunction through an active crosstalk between the two cell types.

### 3.2. Vascular Cells: Endothelial and Smooth Muscle Vascular Cells

**Figure 2:**
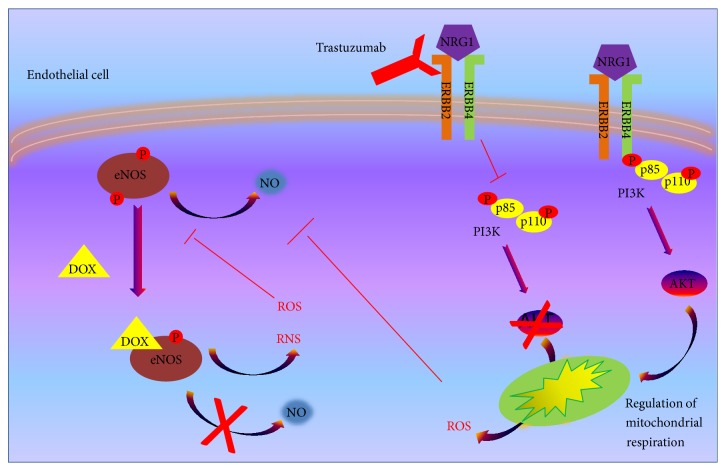
Effects of anticancer drugs that induce oxidative stress on endothelial cells. Trastuzumab inhibits HER2/4 pathway, blocking activation of AKT and its regulatory effect on mitochondrial respiration, with consequent ROS accumulation. The latter causes the inhibition of eNOS activity. DOX, is able to directly bind eNOS, inhibiting NO production and inducing RNS and ROS accumulation.

The ability of endothelial cells to regulate the vascular tone and the absorption of metabolites and drugs, together to their paracrine action on the other cardiac cells through the active release of several factors, makes these cells key mediators of cardiotoxic response. Indeed, several cardiotoxic drugs affect endothelial function by inducing oxidative stress [56]. It has been shown in endothelial cells that DOX administration increases cellular permeability, leading to edema formation that characterizes animals and humans treated with anthracycline [57]. Moreover, in the same cells, DOX reduces ATP and glutathione (GSH), suggesting that the treatment with DOX depletes the cells of their antioxidant capacity and induces mitochondrial dysfunction [56]. The toxic effect of DOX in endothelial cells was also derived from its ability to react with NO synthase, blocking NO production, which has a central role in endothelial homeostasis (Figure 2). Besides its effect on endothelial cells, DOX also affects vascular smooth muscle cells (VSMC) leading to a senescence phenotype and impairment of contractility. This latter effect is the result of a downregulation of *α*-adrenergic receptors and the increase of oxidative stress. Indeed, adrenoceptor expression and vessel contraction are partially restored in the presence of SOD, an enzyme actively involved in neutralization of superoxide [58].

An important endothelial injury is also mediated by cyclophosphamide, which affects endothelial integrity resulting in extravasation of proteins, blood cells, and toxic metabolites, and induces a direct damage on the myocardium, resulting in arrhythmias and heart failure [59].

The involvement of endothelial dysfunction in cardiotoxic response to cisplatinum is suggested by studies in animal models. In particular, the treatment with cisplatinum, by infusion of the rat mesenteric artery, induces severe endothelial injury with vacuolation and subendothelial edema and significant reduction of superoxide dismutase; all these effects are minimal when the animals are treated with the antioxidant vitamin E, thus proving the key role of ROS in endothelial toxicity induced by cisplatinum [60]. Another chemotherapeutic agent, 5-fluorouracil, also determinates ROS-dependent endothelial damage. The role of 5-fluorouracil in inducing cardiotoxicity by endothelial injury is suggested by data from patients. In fact, the percentage of incidence of cardiac events in response to 5-fluorouracil is about 7,6%, including acute coronary syndromes, which is likely caused by endothelial dysfunction [61]. The pathophysiology is not clear, but an *in vivo* study shows that animals treated with 5-fluorouracil are characterized by vascular endothelial damage, that can be prevented by probucol, a powerful antioxidant [62].

Endothelial injury is involved also in cardiotoxicity induced by novel biological chemotherapeutics, such as inhibitor of Her2 (trastuzumab) and anti-angiogenetic compounds (sorafenib). In cardiac endothelial cells, Her2 stimulation by its ligand, NRG1, induces activation of AKT that is able to modify mitochondrial respiration blocking ROS production. The inhibition of Her2 by trastuzumab deprives the endothelium of this protective mechanism, explaining the ROS-mediated toxic effect of this drug on endothelial cells [63, 64]. Moreover, Her2 inhibition by trastuzumab seems to affect also NO production, with further impairment of endothelial function [65] (Figure 2). It is known that inhibitors of angiogenesis, such as sorafenib, induce ROS-dependent endothelial dysfunctions even if the molecular mechanisms are not clear yet [66, 67]. It is suggested that the inhibition of angiogenesis could trigger activation of endothelin-1, that could, in turn, affect oxidative stress [68].

Several intracellular proteins are able to affect endothelial function by modulating ROS production, even if their role in response to anticancer drugs has not been elucidated yet. Among them, G-protein coupled receptor kinase 2 (GRK2) is known to favor the development of cardiovascular diseases [69], and recently, it has also been recognized as a modulator of mitochondrial function, including ROS production [70, 71]. It is a “stress molecule,” that, in response to cellular stress, rapidly moves within the different cellular compartments to activate specific pathways [72]. It has been shown that GRK2 removal compromises vascular phenotype and integrity by increasing endothelial ROS production [73]. Thus, it is likely to believe that such kinase could be a potential target of cardiotoxic drugs which triggers ROS production and endothelial dysfunction to induce cardiac damage.

### 3.3. Immune Cells

The influence of both resident and infiltrating immune cells in the heart is fundamental in pathological cardiac remodeling. Also, in cardiotoxic response, the contribution of immune cells is relevant, since the high oxidative stress can trigger cardiac inflammatory status. Indeed, in hypertensive rats, DOX treatment induces an increase of dendritic cells, suggesting that DOX-induced damage in cellular membranes could stimulate the immune response, which is prevented by the pretreatment with an antioxidant, dexrazoxane [74]. In particular, several studies demonstrate that DOX induces the innate immune response. In fact, TLR4-deficient mice, after exposure to DOX, show reduced oxidative stress and inflammatory status and an amelioration of cardiac function [75]. The same occurs in TLR2 KO mice (TLR2) [75]. Therefore, cardiotoxic anticancer drugs induce a condition known as “sterile inflammation.” Little is known about this phenomenon, but it seems to start with the activation of inflammasomes, multiprotein complexes that regulate the release of proinflammatory cytokines. Among them, NLRP3 inflammasome seems to be the one implicated in sterile inflammation [76, 77]. It has been shown that DOX is able to activate NLRP3 inflammasome, inducing the release of IL-1*β* in macrophages *in vitro* [78]. Accordingly, in mice exposed to DOX, there is an increase in serum levels of IL-1*β* and other inflammatory factors (CCL2/MCP-1) [78]. Moreover, the treatment with the IL-1*β* receptor blocker protects against DOX cardiotoxicity [79].

Thus, it is clear that DOX is able to induce an inflammatory status but the mechanisms by which it regulates inflammasome activation is still unknown. Given the ability of DOX to induce ROS production, it is likely that such phenomenon could depend on oxidative stress. Indeed, DOX-dependent inflammation is prevented in presence of ROS inhibitors, N-acetyl-cysteine, and diphenyl iodonium [78]. Moreover, the inflammasome can be activated by mitochondrial ROS, which are produced by macrophages in response to different stimuli, including angiotensin II [80, 81]. The correlation between cardiotoxicity, inflammation, and oxidative stress also emerges from clinical data. Indeed, in patients treated with DOX, high serum levels of proinflammatory factors (IL-6, TNF, and its soluble receptors, sIL-6R) are detected and correlated with levels of ROS, antioxidant enzymes, and markers of heart dysfunction (CK-MB, BNP) [82].

Similarly, also trastuzumab and sunitinib are able to induce inflammatory responses in cardiac tissue [83, 84]. In sunitinib-treated rats, for instance, high NF*κ*B mRNA levels and accumulation of profibrotic factors and a NOX2 subunit of NADPH oxidase were detected [84].

Thus, all these findings suggest a hypothetical mechanism by which inflammation is activated in heart tissue in response to anticancer drugs. Likely, drugs induce ROS production with consequent cell membrane damage, apoptosis, and necrosis which activate the process of antigen presentation. This mechanism contributes to the activation of resident immune cells and to the infiltration of circulating immune cells, triggering inflammatory response through inflammasome activation. Besides inflammasome activation, the inflammatory status is also sustained by the activation of several transcription factors, such as NF*κ*B, PPAR-*γ*, p53, and HIF-1*α*, which favor the expression of inflammatory genes [85, 86]. Moreover, immune cells, in turn, produce a “respiratory burst,” due to the increase of oxygen uptake, which determinates further ROS production in the site of damage [87, 88]. Also, in these cell lines, the potential role of GRK2 could be of great interest. Indeed, in RAW267.4, GRK2 levels are increased in response to inflammatory stimuli and the kinase is mainly localized in the mitochondria where it regulates ROS production [81]. Moreover, GRK2 is able to activate NF*κ*B signaling in the heart to induce cardiac hypertrophy [69]. These findings strongly suggest that this kinase could be involved in the crosstalk between cardiac immune cells and myocytes and could be a potential target of cardiotoxic agents.

### 3.4. Cardiac Progenitor Cells

Several pieces of evidence demonstrate that the adult heart of humans and animals contains a pool of progenitor cells (cardiac progenitor cells (CPC)) which are able to differentiate in myocytes and coronary vessels [89–91]. These cells have a relevant function during the response to cardiac damage, mediating tissue repair and regeneration, but are more sensitive to oxidative stress than myocytes, rapidly inducing apoptosis [92], suggesting that they could be the primary target of cardiotoxic drugs. In a model of DOX-induced cardiomyopathy, a significant reduction of viable CPCs occurs [92]. Moreover, if the implantation of healthy CPCs determines cardiac functional recovery, the use of DOX-treated CPCs does not provide benefits [93]. The effects of DOX on CPCs are multiple: oxidative DNA damage, which in turn affects cellular cycle, apoptosis, senescence pathways, prosurvival pathways, and antioxidant capabilities of CPCs. Indeed, DOX-treated CPCs are characterized by a significant increase of 8-OH-deoxyguanosine in nuclei and an increased expression of phosphorylated form of histone H2, markers of ROS-dependent DNA damage [92, 94]. Moreover, an increase of p-ATM (Ser1981) and p-p53 (Ser15), involved in cell cycle arrest and apoptosis, also occur [94, 95]. The increase of cell cycle inhibitor p16-INK4 together with the reduction of telomerase activity in DOX-treated CPCs triggers the cellular senescence pathways [95, 96]. These data are confirmed by studies in human which show that in patients with anthracycline-induced cardiomyopathy, there is a significant increase of senescent CPCs characterized by accumulation of p16 INK4 compared with age-matched controls [94, 97]. Besides these effects on oxidative DNA damage, it has also been demonstrated that DOX reduces the expression of IGF-1R, affecting the activation of prosurvival pathways [94, 98] and C-Met, compromising the migration of CPCs in the site of cardiac damage [94, 99]. Doxorubicin also affects the antioxidant capacities of CPCs by affecting the activity of MnSOD, Cu/ZnSOD, and catalase [92]. All these effects on CPCs trigger a latent cardiac damage that arises only in response to stress conditions. Indeed, the treatment with DOX in mice in juvenile age induces a reduction of CPCs and accumulation of senescence markers without apparent morphological and functional cardiac alterations. However, these mice in adult age are more sensitive to physiological and pathological stress (exercise, myocardial infarction) [96].

Less is known about the effects on CPCs of other cardiotoxic drugs. The ability of cyclophosphamide to affect cardiac differentiation from primitive cells has been tested only in embryonic cardiac stem cells and not in adult CPCs, showing that embryonic cardiac precursors are more sensitive to cyclophosphamide than late mature cardiomyocytes [100]. It has been shown that 5-fluorouracil (5-FU) induces depletion of cardiac stem cells with drastic reduction of cardiac neogenesis but the exact mechanism is still unknown [101]. The effects of trastuzumab on CPCs have been tested only in *in vitro* studies showing that the drug does not affect apoptosis and cell viability but reduces the ability to differentiate in cardiomyocytes and to form microvascular networks [102]. Moreover, animals with myocardial infarction treated with CPCs and trastuzumab does not show a recovery of cardiac phenotype compared with the animals treated only with CPCs [102].

## 4. Cardiac Cell Crosstalk in Cardiotoxicity

**Figure 3:**
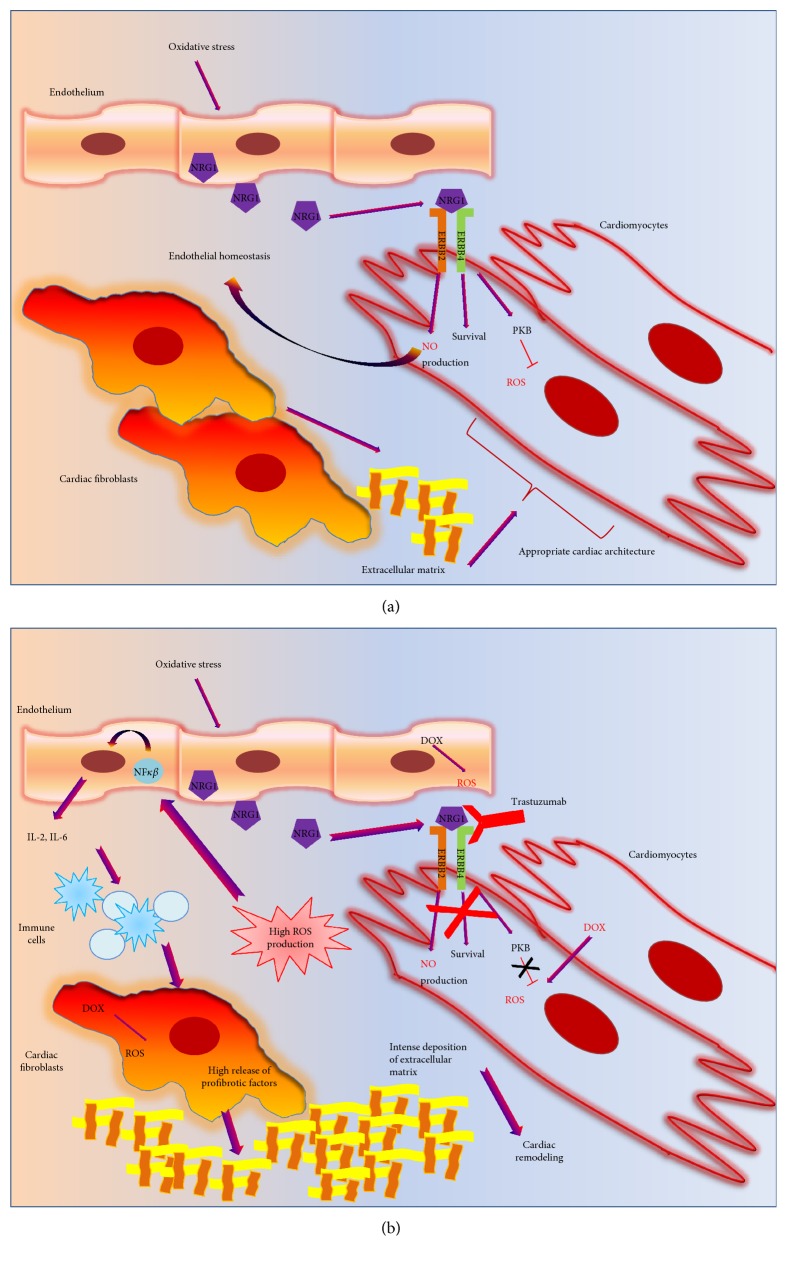
A functional crosstalk between cardiac cells, affecting by cardiotoxic drugs. (a) In response to physiological stress, endothelial cells secrete survival factors, NRG1. NRG1 acts on HER2/HER4 in cardiomyocytes, inducing survival pathways, blocking ROS production, and favoring NO release. Cardiac fibroblasts produce components of extracellular matrix to ensure appropriate heart architecture also in response to changes in cardiac homeostasis. (b)The defensive mechanism mediated by NRG1 does not occur in presence of trastuzumab. Moreover, the oxidative stress induced by anticancer drugs, such as DOX or trastuzumab, induces activation of NF*κ*B in the endothelium, with an expression of proinflammatory cytokines, IL-2 and IL-6, activating immune cells. The activated immune cells promote a profibrotic phenotype of fibroblasts, with abnormal deposition of extracellular matrix and pathological cardiac remodeling.

Cardiotoxicity is a very complex phenomenon that is the result of heart damage in toto, including all the different cardiac cells (endothelial, cardiomyocytes, fibroblast, and immune cells). Although these cells are different in metabolism, function, and structure, they are part of a complex network in which they participate in active crosstalks. Thus, each cell type is able to change its functional features (metabolic demand, oxygen consumption, migration, and secretion) to maintain tissue homeostasis. To date, this phenomenon is still poorly explored, even if several pieces of evidence sustain the existence of crosstalks between cardiac cells in both physiologic and pathologic conditions [103]. The effects of anticancer drugs on this phenomenon are different (activation or inhibition of cell crosstalks) due to the type of drug and affected pathway. Generally, in response to oxidative stress, cardiac endothelial cells release neuregulin, a peptide that activates cardiomyocyte receptors HER4, which in turn dimerizes with HER2 receptors and activates survival pathways (inhibition of apoptosis and reduction of mitochondrial ROS generation through activation of protein kinase B) [63] (Figure 3(a)). Moreover, HER4/HER2 signaling also increases NO production, which in turn acts on the endothelium to preserve endothelial homeostasis [64]. This interplay between cardiomyocytes and endothelial cells, that represents a defensive mechanism during the stress response, does not occur in the presence of trastuzumab (Figure 3(b)). Thus, it appears clear that the effects of anticancer treatment on cardiac function are amplified by the disruption of cardiac cell crosstalks.

Also, anthracyclines are able to regulate cell crosstalks. Indeed, DOX-dependent oxidative stress in cardiac fibroblast induces the release of FAS-L which in turn activates apoptosis signaling in cardiomyocytes [104].

Moreover, the inflammatory response to cardiotoxic drugs is itself the result of an active interplay between cardiac cells. Indeed, the oxidative stress induced by cardiotoxic drugs activates NF*κ*B in the endothelium with consequent release of proinflammatory cytokines (IL-1, IL-2, and IL-6) which, in turn, acts in resident and circulating immune cells. The latter affect the function of fibroblasts, inducing deposition of fibrotic tissue that influence contractile function of cardiomyocytes (Figure 3(b)).

The mechanisms of cell interplay within the heart are very complex, and further investigations are needed to understand their involvement in the suiting of cardiotoxicity. This could be the main target of next future studies which will allow in this manner the identification of new therapeutic strategies, based on the targeting of molecules that mediate cell communications.

## 5. Future Prospective

To date, cardiotoxicity is a rising issue for several oncologic patients which undergo chemotherapy to treat cancer. This is due to the cardiotoxic effects of some chemotherapeutic agents that although effective to increase patient's survival to cancer, in most cases lead to patient's death for cardiovascular diseases. This suggests the need for new strategies to prevent or treat cardiotoxicity. Given the key role of ROS in determining the cardiotoxic response, a therapeutic strategy based on the use of antioxidant could be useful. Also, given the important contributions of the different cardiac cells in cardiotoxicity, cell-specific molecular targets could be identified to prevent oxidative stress and the associated cell damage. In this context, the specific inhibition of the GRK2 activity or the regulation of its subcellular localization, that has been shown to be an effective strategy for the treatment of cardiovascular diseases, could also be useful in the treatment of cardiotoxicity, even if further studies are needed to sustain this hypothesis.

## Figures and Tables

**Table 1:** List of anticancer drugs that exert direct or indirect effects on ROS production.

Direct effects	Indirect effects
Anthracyclines	Taxanes
Cyclophosphamide	Trastuzumab
5-Fluorouracil	Sorafenib
Cisplatinum	

**Table 2:** Summary of major pathways affected by cardiotoxic drugs within the different cardiac cell types.

Type of cell	Drug	Molecular pathway
Cardiac fibroblasts	Doxorubicin	(i) Activation of TGF-*β* and collagen deposition (ii) Activation of ATM (iii) Release of Fas-L and activation of apoptosis
	Cobalt	(i) Reduction of MnSOD and reduced ROS scavenger capacity

Endothelial cells	Doxorubicin	(i) Increase of cellular permeability (ii) Reduction of GSH, MnSOD, and reduced ROS scavenger capacity (iii) Reduction of ATP and mitochondrial dysfunction (iv) Inhibition of NO synthase (v) Activation of NF*κ*B and release of IL-1/IL-2/IL-6
	Cyclophosphamide	(i) Increase of cellular permeability (ii) Activation of NF*κ*B and release of IL-1/IL-2/IL-6
	Cisplatinum	(i) Reduction of GSH, MnSOD, and reduced ROS scavenger capacity (ii) Activation of NF*κ*B and release of IL-1/IL-2/IL-6
	Trastuzumab	(i) Inhibition of NO synthase
	Sorafenib	(i) Activation of endothelin 1

Vascular smooth muscle cells	Doxorubicin	(i) Activation of senescence (ii) Downregulation of *α*-adrenoreceptor and reduction of contractility

Immune cells	Doxorubicin	(i) Activation of innate immune response (ii) Activation of NLRP3 inflammasome and release of IL-1*β* and MCP-1
	Trastuzumab	(i) Increase of NF*κ*B expression (ii) Increase of NADPH subunit levels
	Sunitinib	(i) Increase of NF*κ*B expression (ii) Increase of NADPH subunit levels

Cardiac progenitor cells	Doxorubicin	(i) Oxidative DNA damage and activation of ATM and p53 (ii) Increase of p16 INK4 and activation of senescence (iii) Reduction of IGF-1 and c-Met with reduction of survival and cell migration (iv) Reduction of MnSOD, Cu/Zn SOD, catalase, and reduced ROS scavenger capacity (v) Increase of apoptosis and inhibition of cardiac differentiation
	5-Fluorouracil	(i) Increase of apoptosis and inhibition of cardiac differentiation
	Trastuzumab	(i) Increase of apoptosis and inhibition of cardiac differentiation
